# Role of autophagy in drug induced liver injury

**DOI:** 10.1007/s00204-020-02887-z

**Published:** 2020-08-27

**Authors:** Tim Brecklinghaus

**Affiliations:** Leibniz Research Centre for Working Environment and Human Factors, Ardeystr. 67, 44139 Dortmund, Germany

Recently, Wandrer and colleagues published a study about the interaction of endoplasmic reticulum (ER) stress and autophagy and its relevance for hepatotoxicity (Wandrer et al. 2020). For this purpose, they used the iodinated benzofurane derivative amiodarone as a model compound. Amiodarone is known to induce steatohepatitis (Lewis et al. 1989; Vassallo and Trohman 2007) of which a relatively high fraction progresses to liver cirrhosis (Rigas 1989; Farrell 2002; Raja et al. 2009). Amiodarone mediated DILI has been reported to progress despite of discontinuation of the drug, possibly because of the very long half-life in the liver (Lewis et al. 1989; Vassallo and Trohman 2007; Chang et al. 1999; Brien et al. 1987). In the present study, Wandrer and colleagues demonstrated that patients treated with amiodarone had higher serum levels of the apoptosis marker caspase-cleaved keratin-18 compared to individuals not treated with amiodarone. Next, they demonstrated that incubation of hepatocytes with amiodarone induced lipid accumulation and ER-stress (Wandrer et al. 2020). Interestingly, co-incubation of amiodarone with the autophagy inhibitor chloroquine increased amiodarone-induced toxicity. Also, in ATG5- or ATG7-deficient hepatocytes amiodarone-triggered toxicity was increased.

Drug-induced liver injury represents a major research field in toxicology (Godoy et al. 2013; Leist et al. 2017; Jansen et al. 2017). Research aims at a better understanding of DILI mechanisms (Albrecht et al. 2019; Vartak et al. 2016; Reif et al. 2017) and the establishment of in vitro (Gu et al. 2018; Grinberg et al. 2014, 2018; Frey et al. 2014) and in silico methods (Ghallab et al. 2016, 2019; Hoehme et al. 2010). Nevertheless, still relatively little is known how autophagy modifies DILI and if in vitro findings are also relevant to the in vivo situation. Wandrer and colleagues are to be congratulated, because they not only demonstrate that autophagy induction ameliorates amiodarone mediated hepatotoxicity but also study human in vivo relevance by analysis of the apoptosis marker keratin-18 in serum.
